# Involvement of Ca^2+^ Signaling in the Synergistic Effects between Muscarinic Receptor Antagonists and β_2_-Adrenoceptor Agonists in Airway Smooth Muscle

**DOI:** 10.3390/ijms17091590

**Published:** 2016-09-21

**Authors:** Kentaro Fukunaga, Hiroaki Kume, Tetsuya Oguma, Wataru Shigemori, Yuji Tohda, Emiko Ogawa, Yasutaka Nakano

**Affiliations:** 1 Division of Respiratory Medicine, Department of Internal Medicine, Shiga University of Medical Science, Seta, Tsukinowa-cho, Otsu, Shiga 520-2192, Japan; fukuken@belle.shiga-med.ac.jp (K.F.); oguma@belle.shiga-med.ac.jp (T.O.); nitori@belle.shiga-med.ac.jp (W.S.); eogawa@belle.shiga-med.ac.jp (E.O.); nakano@belle.shiga-med.ac.jp (Y.N.); 2 Department of Respiratory Medicine and Allergology, Kindai University Faculty of Medicine, 377-2 Ohnohigashi, Osakasayama, Osaka 589-8511, Japan; tohda@med.kindai.ac.jp

**Keywords:** cross talk, Ca^2+^ dynamics, Ca^2+^ sensitization, large-conductance Ca^2+^-activated K^+^ channel, protein kinase C, tracheal smooth muscle, tiotropium, procaterol, COPD

## Abstract

Long-acting muscarinic antagonists (LAMAs) and short-acting β_2_-adrenoceptor agonists (SABAs) play important roles in remedy for COPD. To propel a translational research for development of bronchodilator therapy, synergistic effects between SABAs with LAMAs were examined focused on Ca^2+^ signaling using simultaneous records of isometric tension and F340/F380 in fura-2-loaded tracheal smooth muscle. Glycopyrronium (3 nM), a LAMA, modestly reduced methacholine (1 μM)-induced contraction. When procaterol, salbutamol and SABAs were applied in the presence of glycopyrronium, relaxant effects of these SABAs are markedly enhanced, and percent inhibition of tension was much greater than the sum of those for each agent and those expected from the BI theory. In contrast, percent inhibition of F340/F380 was not greater than those values. Bisindolylmaleimide, an inhibitor of protein kinase C (PKC), significantly increased the relaxant effect of LAMA without reducing F340/F380. Iberiotoxin, an inhibitor of large-conductance Ca^2+^-activated K^+^ (K_Ca_) channels, significantly suppressed the effects of these combined agents with reducing F340/F380. In conclusion, combination of SABAs with LAMAs synergistically enhances inhibition of muscarinic contraction via decreasing both Ca^2+^ sensitization mediated by PKC and Ca^2+^ dynamics mediated by K_Ca_ channels. PKC and K_Ca_ channels may be molecular targets for cross talk between β_2_-adrenoceptors and muscarinic receptors.

## 1. Introduction

Chronic obstructive pulmonary disease (COPD) is characterized by irreversible narrowing of the small airways and decreased elastic recoil of the lung due to destruction of the alveoli [1,2]. As a result, hyperinflation occurs, causing dyspnea during exercise, and decreasing exercise tolerability in patients with COPD [3,4]. Although the fundamental pathophysiology of COPD is considered to be neutrophilic inflammation in the airways, there are currently no specific anti-inflammatory agents for this disease. Inhaled bronchodilators, such as muscarinic receptor antagonists and β_2_-adrenoceptor agonists, are widely used clinically to reduce symptoms (shortness of breath, wheezing), augment lung function (forced expiratory volume in 1 second, inspiratory capacity), and reduce exacerbations and hospitalizations, improving quality of life.

Inhalation of muscarinic receptor antagonists [5,6] and β_2_-adrenoceptor agonists [7,8] is used in the management of COPD. However, a single type of bronchodilator may be insufficiently effective for this disease. A recent COPD guideline states that a combination of bronchodilators of different pharmacological classes may improve effectiveness and decrease the risk of adverse reactions compared to increasing the dose of a single bronchodilator [2]. Long-acting β_2_-adrenoceptor agonists (LABAs) and long-acting muscarinic receptor antagonists (LAMAs) are two types of bronchodilators widely used as therapy for this disease, and there is a pharmacological rationale for the use of a combination of these agents [9,10,11,12]. Recent studies in patients with COPD have also demonstrated that the LABA/LAMA combination is more effective in improving symptoms and lung function, and reducing exacerbations, than monotherapy with either agent [13,14,15,16].

**Figure 1:**
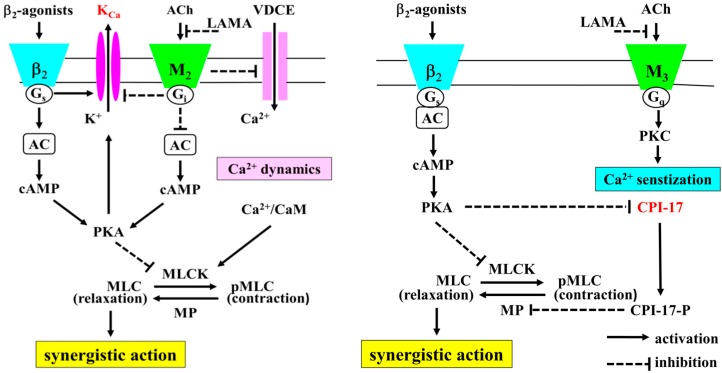
The intracellular mechanisms of the synergistic effects between β_2_-adrenoceptor agonists and muscarinic receptor antagonists. Not only Ca^2+^ sensitization but also Ca^2+^ dynamics contributes to the synergistic effect of β_2_-adrenoceptor agonists and muscarinic receptor antagonists (cross talk between β_2_-adrenoceptors and muscarinic receptors). In Ca^2+^ dynamic, Ca^2+^ influx by VDCE is involved in this phenomenon. VDCE is regulated by membrane potential via K_Ca_ channel activity, which is augmented by G_s_ coupled to β_2_-adrenoceptor, in contrast, attenuated by G_i_ coupled to muscarinic M_2_ receptors (dual regulation by G proteins). Increased intracellular Ca^2+^ concentration causes contraction by activation of MLCK via Ca^2+^/CAM processes (Ca^2+^-dependent contraction). In Ca^2+^ sensitization, PKC is involved in this phenomenon. PKC is activated by muscarinic M_3_ receptors. PKC inhibits MP activity via CPI-17 processes. Inactivation of MP causes contraction by increased sensitivity to intracellular Ca^2+^ (Ca^2+^-independent contraction). CPI-17 MP is activated by PKC, in contrast, inhibited by PKA. K_Ca_ channels and CPI-17 are key molecules for this synergistic effect via the cross talk between these two receptors. This synergism may be caused by Ca^2+^ dynamics (tone with changes in concentration of intracellular Ca^2+^) via K_Ca_ channel activity reciprocally regulated by G proteins (G_s_ and G_i_), and caused by Ca^2+^ sensitization (tone without changes in concentration of intracellular Ca^2+^) via CPI-17 reciprocally regulated by protein kinases (PKA and PKC). K_Ca_: large-conductance Ca^2+^-activated K^+^ channels. VDCE: voltage-dependent Ca^2+^ entry. ACh: acetylcolin, LAMA: long-acting muscarinic receptor antagonist. AC: adenylate cyclase. PKA: protein kinase A. PKC: protein kinase C. CPI-17: C-kinase-potentiated protein phosphatase-1 inhibitor. CaM: calmodulin. MLCK: myosin light chain kinase. MP: myosin phosphatase.

In airway smooth muscle, LABAs and LAMAs act synergistically, enhancing inhibition of muscarinic contraction more than the sum of the effects of each agent [9,10]. Cross talk between these two receptors may contribute to this synergistic effect in airway smooth muscle. Large-conductance Ca^2+^-activated K^+^ (K_Ca_) channels are regulated by G_s_ proteins coupled to β_2_-adrenoceptors [17,18,19] and G_i_ proteins coupled to muscarinic M_2_ receptors [18,19,20,21]. Since the functional antagonism between β_2_-adrenoceptor and muscarinic receptor action converges on K_Ca_ channels [22], this synergism may be caused by Ca^2+^ dynamics (changes in concentration of intracellular Ca^2+^) via K_Ca_ channel activity (Figure 1) [23,24]. However, little is currently known about the involvement of Ca^2+^ sensitization in the mechanism underlying this synergy between these two G protein-coupled receptors (GPCRs), such as β_2_-adrenoceptors and muscarinic receptors.

Various contractile agents such as acetylcholine cause smooth muscle contraction via GPCRs. Airway smooth muscle tone is regulated by Ca^2+^ dynamics and Ca^2+^ sensitization. The Ca^2+^ dynamics is a mechanism of airway smooth muscle contraction via activation of a Ca^2+^/calmodulin-dependent myosin light chain kinase and increase intracellular Ca^2+^ concentration via a Ca^2+^ influx through Ca^2+^ channels (Ca^2+^-dependent contraction). In contrast, since Phosphorylation (inactivation) of myosin phosphatase (MP) causes contraction as intracellular Ca^2+^ level unchanged, MP inhibition contributes to Ca^2+^ sensitization (Ca^2+^-independent contraction) [25]. MP is phosphorylated by Rho-kinase, which is an effector of the small G protein RhoA [26,27,28] and by C-kinase-potentiated protein phosphatase-1 inhibitor (CPI-17), which is another potential mediator regulated by protein kinase C (PKC) (Figure 1) [29,30,31]. Ca^2+^ signaling in airway smooth muscle plays an essential role in the pathophysiology of asthma and COPD [9,10,32,33,34,35,36,37].

Inhalation of rapid- and short-acting bronchodilators such as short-acting β_2_-adrenoceptor agonists (SABAs) is useful for symptom relief in patients with COPD. Because of the synergistic effect of these two agents on bronchodilation, pre-administration of LAMAs may be effective for as-needed use of SABAs, such as salbutamol and procaterol. Therefore, there is a need for the development of pharmacological treatments for COPD that exploit the synergistic effects between LAMAs and SABAs. This study was designed to determine whether procaterol and salbutamol (SABAs) and glycopyrronium (a LAMA) act synergistically to relax contracted airway smooth muscle. We promoted this study on the assumption of involvement of the major pathway in Ca^2+^ dynamics and Ca^2+^ sensitization, which play a key role of contraction of airway smooth muscle. In particular, mechanisms underlying this phenomenon were examined with a focus on Ca^2+^ signaling, specifically Ca^2+^ dynamics mediated by K_Ca_ channels and Ca^2+^ sensitization mediated by Rho-kinase and PKC in airway smooth muscle. The functional antagonism between β_2_-adrenergic and muscarinic action converges on K_Ca_ channel currents, and Rho-kinase and PKC play key role in muscarinic contraction of airway smooth muscle.

## 2. Results

### 2.1. Inhibitory Effects of Procaterol, Salbutamol, and Glycopyrronium on Tension and Intracellular Ca^2+^ Concentration in Contracted Muscle

**Figure 2:**
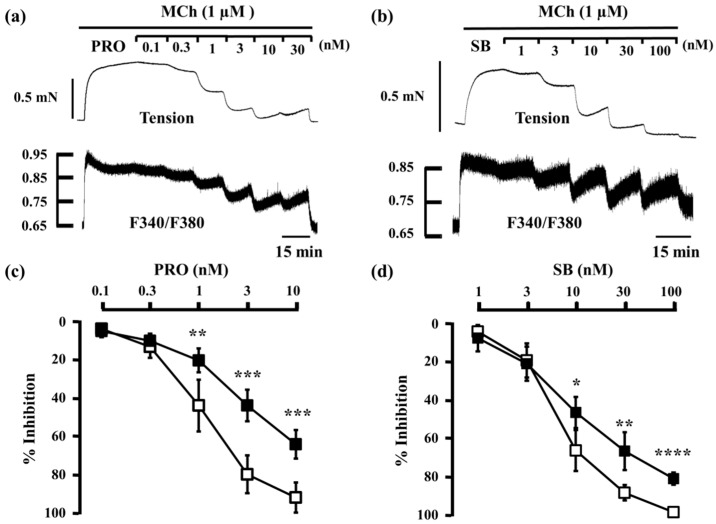
Involvement of Ca^2+^ dynamics and Ca^2+^ sensitization in the relaxant effect of β_2_-adrenoceptor agonists. (**a**,**b**) Typical samples of continuous recording of tension and F340/F380 demonstrating the inhibitory effect of procaterol (0.1–30 nM) (**a**) and salbutamol (1–100 nM) (**b**) on MCh (1 µM)-induced smooth muscle contraction; (**c**,**d**) Concentration–response curve for procaterol (0.1–10 nM) (**c**) and salbutamol (1–100 nM) (**d**) in tension (□) and F340/F380 (■) in 1 µM MCh-precontracted smooth muscle. Resting state tension and F340/F380 were taken as 0%, and those in each MCh-stimulated state were taken as 100%. MCh, methacholine; PRO, procaterol; SB, salbutamol. **** *p* < 0.0001; *** *p* < 0.001; ** *p* < 0.01; * *p* < 0.05.

**Figure 3:**
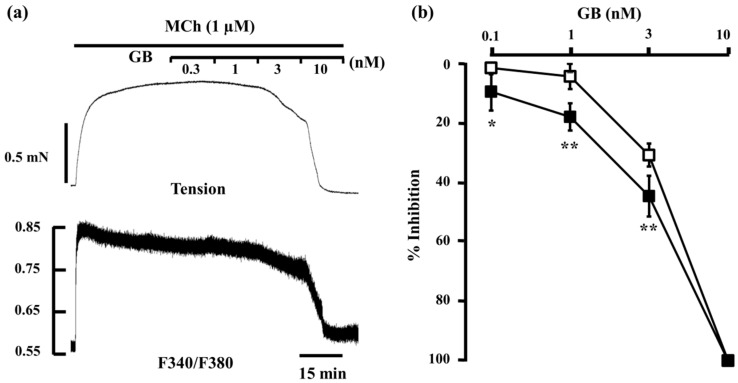
Involvement of intracellular Ca^2+^ dynamics in the relaxant effect of glycopyrronium. (**a**) Typical sample of continuous recording of tension and F340/F380 demonstrating the inhibitory effect of glycopyrronium (0.3–10 nM) on MCh (1 µM)-induced contraction; (**b**) Concentration–response curve for glycopyrronium (0.1–10 nM) in tension (□) and F340/F380 (■) induced by MCh (1 µM). GB, glycopyrronium bromide; MCh, methacholine. ** *p* < 0.01; * *p* < 0.05.

When procaterol (0.1–10 nM) and salbutamol (1–100 nM) were cumulatively applied to strips of tracheal smooth muscle pre-contracted with MCh (always 1 μM throughout this study), both inhibited MCh-induced contraction and reduced F340/F380 in a concentration-dependent manner. However, when almost complete relaxation was achieved at the maximum concentrations tested (procaterol, 10 nM; SB, 100 nM), F340/F380 was still higher than at the basal level (Figure 2a,b). The concentration–inhibition curve of procaterrol and salbutamol for MCh-induced tension was significantly dissociated from the curve for F340/F380 (*n* = 5, *p* < 0.05), and the reduction in tension was significantly greater than the reduction in F340/F380 at each concentration ≥1 nM (procaterol) and ≥10 nM (salbutamol) (Figure 2c,d). However, when glycopyrronium (0.3–10 nM) was cumulatively applied to the strips of tracheal smooth muscle in the same way, glycopyrronium inhibited MCh-induced contraction and reduced F340/F380 in a concentration-dependent manner. The concentration–inhibition curve of glycopyrronium for MCh-induced tension was also dissociated from the curve for F340/F380 (*n* = 5, *p* < 0.05), but the reduction in F340/F380 was significantly greater than that in tension at each concentration (0.1–3 nM) (Figure 3).

### 2.2. Effects of Procaterol and Salbutamol Combined with Glycopyrronium on Tension and Intracellular Ca^2+^ Concentration in Contracted Muscle 

**Figure 4:**
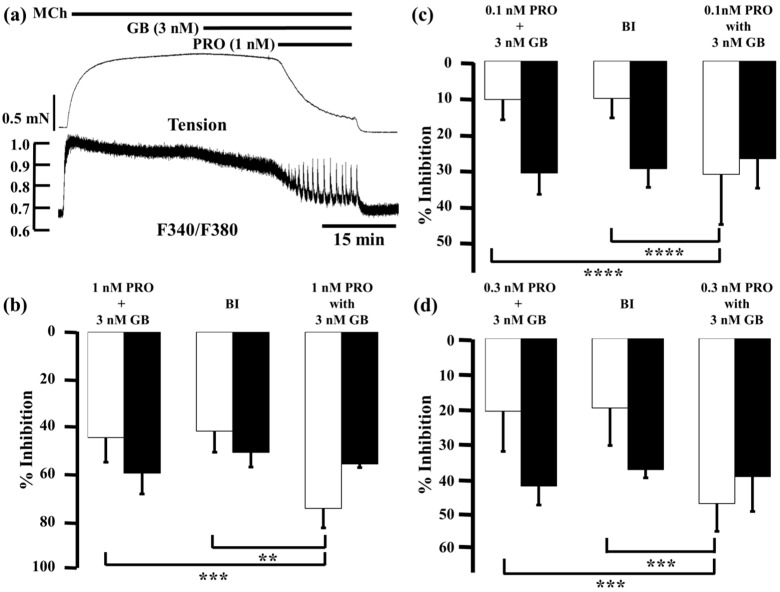
Effects of combination of procaterol and glycopyrronium on tension and intracellular Ca^2+^ concentration induced by muscarinic activation. (**a**) Typical traces of tension (**upper side** and F340/F380 (**lower side**) showing the inhibitory effect of procaterol (1 nM) in the presence of glycopyrronium (3 nM) on MCh (1 µM)-induced contraction; (**b**–**d**) Percent inhibition of tension (white columns) and F340/F380 (black columns) in 1 µM MCh-induced contraction under the experimental conditions of 1 nM procaterol (**b**); 0.1 nM procaterol (**c**); and 0.3 nM procaterol (**d**) in the presence of glycopyrronium (3 nM). **Left**, sum of percent inhibition of tension and F340/F380 by the two agents; **Center**, BI, expected percent inhibition of tension and F340/F380 calculated by the Bliss independence theory; **Right**, percent inhibition of tension and F340/F380 with the two agents in combination. PRO, procaterol; GB, glycopyrronium bromide; MCh, methacholine. **** *p* < 0.0001; *** *p* < 0.001; ** *p* < 0.01.

Glycopyrronium (3 nM) reduced MCh-induced tension and F340/F380 by 7.2% ± 4.5% and 23.6% ± 4.0% (*n* = 5), respectively; procaterol (1 nM) reduced them by 37.0% ± 9.1% and 32.9% ± 5.4% (*n* = 5), respectively. When procaterol (1 nM) was applied to the MCh-precontracted strips in the presence of glycopyrronium (3 nM), inhibition of contraction and decrease in F340/380 were markedly enhanced, to 73.9% ± 8.2% and 55.3% ± 1.5% (*n* = 5), respectively (Figure 4a,b). Under these conditions, inhibition of contraction was considerably greater than the sum of the effects of each agent (44.2% ± 10.3%, *n* = 5, *p* < 0.0001; Figure 4b), and the values predicted by the Bliss independence (BI) theory (41.5% ± 8.8%, *n* = 5, *p* < 0.0001; Figure 4b). A detailed discussion of the BI theory can be found in the method section. In contrast, reduction of F340/F380 was not significantly greater than the sum of the effect of each agent (59.1% ± 8.7%, *n* = 5; Figure 4b) or the expected value by BI theory (50.4% ± 6.2%, *n* = 5; Figure 3b). Next, we lowered the concentration of procaterol to 0.1 and 0.3 nM. At 0.1 nM, procaterol inhibited MCh-induced contraction by 3.4% ± 2.4% and reduced F340/F380 by 4.5% ± 3.2% (*n* = 5). However, in the presence of glycopyrronium (3 nM), those values were augmented to 31.2% ± 13.8% and 26.9% ± 8.1% (*n* = 5), respectively (Figure 4c). Under these conditions, percent inhibition of contraction was also much greater than the sum of the values for each agent (10.6% ± 5.6%, *n* = 5, *p* < 0.001; Figure 4c) and the expected value (10.3% ± 5.4%, *n* = 5, *p* < 0.01; Figure 4c). In contrast, percent inhibition of F340/F380 was not increased significantly more than the sum of the values for each agent (30.8% ± 5.9%, *n* = 5; Figure 4c) or the expected values (29.6% ± 5.2%, *n* = 5; Figure 4c). Percent inhibition of contraction and F340/F380 for procaterol (0.3 nM) in MCh-precontracted tissue were 13.1% ± 11.0% and 11.4% ± 3.6% (*n* = 5), respectively. When procaterol (0.3 nM) was applied in the presence of glycopyrronium (3 nM), those values were augmented to 46.0% ± 7.7% and 38.5% ± 9.7% (*n* = 5), respectively (Figure 4d). Percent inhibition of contraction by the two agents in combination was considerably greater than the sum of the values for each agent (20.3% ± 11.2%, *n* = 5, *p* < 0.001; Figure 4d) and the expected value (19.4% ± 10.4%, *n* = 5, *p <* 0.001; Figure 4d). In contrast, percent inhibition of F340/F380 was not significantly greater for the combination than the sum of the values for each agent (41.1% ± 5.3%, *n* = 5; Figure 4d) or the expected value (36.5% ± 2.3%, *n* = 5; Figure 4d).

**Figure 5:**
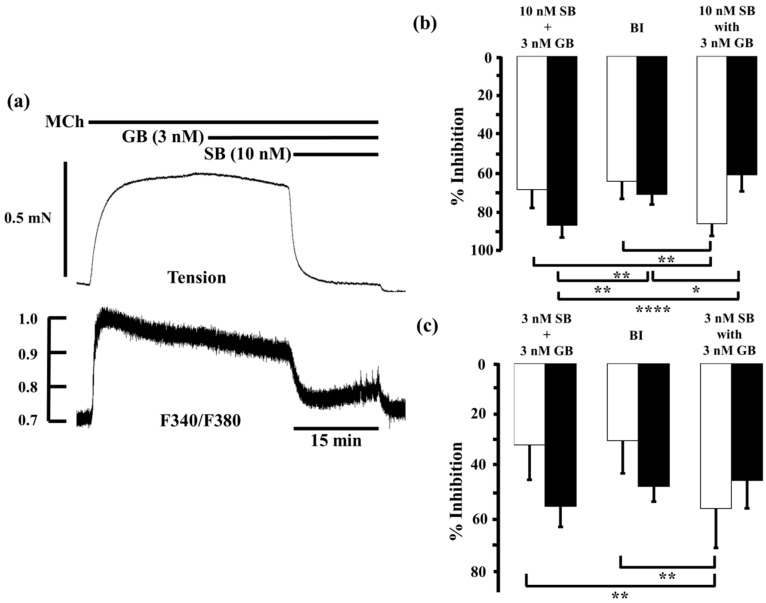
Effects of salbutamol and glycopyrronium in combination on tension and intracellular Ca^2+^ concentration induced by muscarinic activation. (**a**) Typical sample record of tension (**upper side**) and F340/F380 (**lower side**) showing the inhibitory effect of salbutamol (10 nM) in the presence of glycopyrronium (3 nM) against 1 µM MCh-induced contraction; (**b**,**c**) Percent inhibition of tension (white columns) and F340/F380 (black columns) in 1 µM MCh-precontracted tissue incubated with 3 nM salbutamol (**b**) and 10 nM salbutamol (**c**) in the presence of glycopyrronium (3 nM). **Left**, sum of percent inhibition of tension and F340/F380 by the two agents; **Center**, expected percent inhibition of tension and F340/F380 calculated by the Bliss independence theory; **Right**, percent inhibition of tension and F340/F380 with the two agents in combination. SB, salbutamol; GB, glycopyrronium bromide; MCh, methacholine; BI, Bliss independence. **** *p* < 0.0001; ** *p* < 0.01; * *p* < 0.05.

Furthermore, we examined the interaction between glycopyrronium and salbutamol, another SABA, in the same way. Salbutamol (10 nM) inhibited MCh-induced contraction and reduced F340/F380 by 60.8% ± 10.6% and 59.8% ± 8.0% (*n* = 5), respectively. When salbutamol (10 nM) was applied in the presence of glycopyrronium (3 nM), contraction was inhibited by 85.4% ± 6.2% and F340/F380 was reduced by 60.4% ± 8.4% (*n* = 5; Figure 5a,b). The inhibition of contraction by salbutamol (10 nM) combined with glycopyrronium (3 nM) was significantly greater than the sum of the values for each agent (68.0% ± 9.3%, *n* = 5, *p* < 0.01; Figure 5b) and the expected value by BI theory (63.8% ± 8.9%, *p* < 0.01; Figure 4b). However, the combination of agents decreased F340/F380 much by less than the sum of each agent (86.1% ± 6.3%, *n* = 5, *p* < 0.0001; Figure 5b) or the expected value (70.5% ± 5.0%, *p* < 0.05; Figure 5b). The inhibitory effects of salbutamol (3 nM) on these measures were 24.6% ± 14.7% and 29.5% ± 7.0% (*n* = 5), respectively. When salbutamol (3 nM) was applied in the presence of glycopyrronium (3 nM), contraction was inhibited by 56.7% ± 15.3% and F340/F380 was reduced by 45.7% ± 10.9% (*n* = 5; Figure 5c). Contraction was inhibited more than the sum of the effects of each agent (31.8% ± 13.7%, *n* = 5, *p* < 0.01; Figure 5c) and the expected value (30.2% ± 12.7%, *p* < 0.01; Figure 5c), but reduction of F340/F380 was not significantly greater than the sum the effects of each agent (55.8% ± 8.1%, *n* = 5; Figure 5c) or expected value (48.1% ± 5.9%; Figure 5c).

### 2.3. Effects of Procaterol Combined with Tiotropium on Tension and Intracellular Ca^2+^ Concentration in Contracted Muscle

We investigated whether the same synergistic effect was shown when tiotropium administered in combination with procaterol.

**Figure 6:**
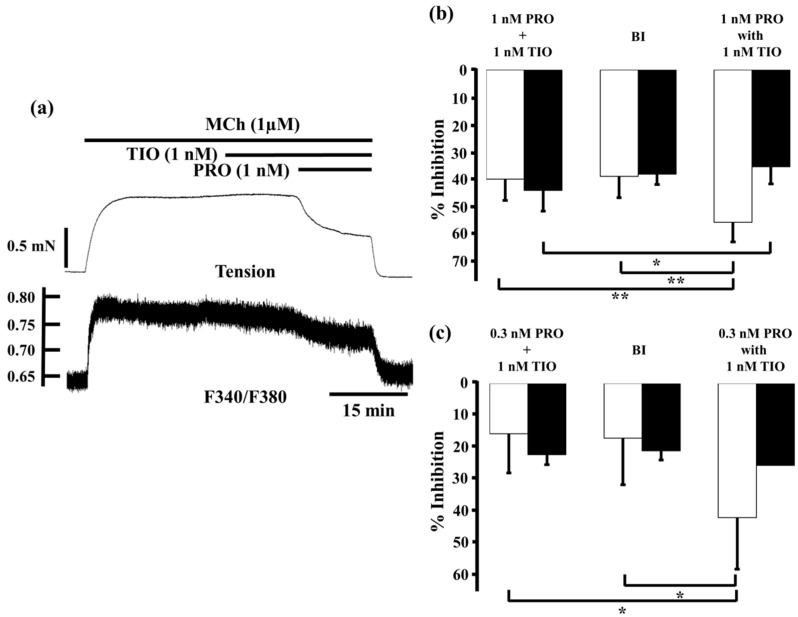
Effects of combination of procaterol and tiotropium on tension and *intracellular Ca^2+^ concentration* induced by muscarinic activation. (**a**) A typical sample record of tension (**upper side**) and F340/F380 (**lower side**) showing the inhibitory effect of procaterol (1 nM) in the presence of tiotropium (1 nM) against 1 µM MCh-induced contraction; (**b**,**c**) Percent inhibition of tension (white columns) and F340/F380 (black columns) in 1 µM MCh-precontracted tissue incubated with 1 nM procaterol (**b**, *n* = 5) and 0.3 nM procaterol (**c**, *n* = 4) in the presence of tiotropium (1 nM). **Left**, sum of percent inhibition of tension and F340/F380 by the two agents; **Center**, expected percent inhibition of tension and F340/F380 calculated by the Bliss independence theory; **Right**, percent inhibition of tension and F340/F380 with the two agents in combination. PRO, procaterol; TIO, tiotropium; MCh, methacholine; BI, Bliss independence. ** *p* < 0.01; * *p* < 0.05.

Tiotropium (1 nM) reduced MCh-induced tension and F340/F380 by 2.9% ± 3.1% and 11.3% ± 3.6% (*n* = 5), respectively. When procaterol (1 nM) was applied to the MCh-precontructed strips in the presence of tiotropium (1 nM), inhibition of contruction and decrease in F340/F380 were markedly enhanced, to 55.6% ± 7.1% and 35.5% ± 5.5% (*n* = 5), respectively (Figure 6a,b). Under these conditions, inhibition of contraction was considerably greater than the sum of the effects of each agent (39.9% ± 7.8%, *n* = 5, *p* < 0.01; Figure 6b), and the expected value (38.9% ± 7.9%, *n* = 5, *p* < 0.01; Figure 6b). In contrast, reduction of F340/F380 was significantly greater than the sum of the effect of each agent (44.1% ± 7.6%, *n* = 5, *p* < 0.05, Figure 6b), but that value was not significant greater than the expected value (38.1% ± 3.8%, *n* = 5, Figure 6b). At 0.3 nM procaterol, in the presence of tiotropium (1 nM), inhibition of contraction and decrease in F340/F380 were augmented to 42.6% ± 16.4% and 26.0% ± 14.4% (*n* = 5, Figure 6c), respectively. Under these conditions, percent inhibition of contraction was also much greater than the sum of the values for each agent (16.0% ± 12.4%, *n* = 4, *p* < 0.05; Figure 6c) and the expected value (17.4% ± 14.8%, *n* = 4, *p* < 0.05; Figure 6c). In contrast, percent inhibition of F340/F380 was not increased significantly more than the sum of the values for each agent (22.6% ± 3.2%, *n* = 4; Figure 6c) or the expected values (21.4% ± 2.9%, *n* = 4).

### 2.4. Role of Ca^2+^ Sensitization in the Combined Effects of Procaterol and Glycopyrronium

From results of our experiments (Figure 4, Figure 5 and Figure 6), synergistic effect of LAMAs and SABAs were involved not only Ca^2+^ dynamics but also Ca^2+^ sensitization. Therefore, we investigated which RhoA/Rho kinase or PKC processes were involved more strongly in this synergistic effect.

**Figure 7:**
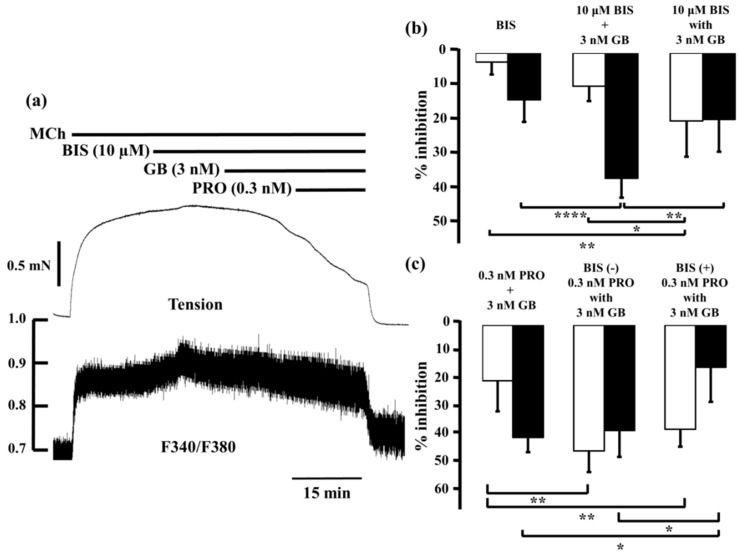
Involvement of protein kinase C-induced Ca^2+^ sensitization in synergy between β_2_-adrenoceptor agonists and muscarinic receptor antagonists. (**a**) Typical record of tension (**upper side**) and F340/F380 (**lower side**) of the inhibitory effect in combination with procaterol (0.3 nM) and glycopyrronium (3 nM) in the presence of bisindolylmaleimide (10 µM) against 1 µM MCh-induced contraction. In each panel, percent inhibition of tension is represented by white columns, and F340/F380 is represented by black columns; (**b**) **Left**, percent inhibition in tension and F340/F380 by bisindolylmaleimide (10 μM); **Center** , sum of percent inhibition of tension and F340/F380 with bisindolylmaleimide (10 µM) and glycopyrronium (3 nM); **Right**, percent inhibition of tension and F340/F380 with combination of bisindolylmaleimide (10 µM) and glycopyrronium (3 nM); (**c**) **Left**, sum of percent inhibition of tension and F340/F380 with procaterol (0.3 nM) and glycopyrronium (3 nM); **Center**, percent inhibition of tension and F340/F380 in combination with procaterol (0.3 nM) and glycopyrronium (3 nM); **Right**, percent inhibition of tension and F340/F380 with combination of procaterol (0.3 nM) and glycopyrronium (3 nM) in the presence of bisindolylmaleimide (10 µM). GB, glycopyrronium bromide; PRO, procaterol; MCh, methacholine; BIS, bisindolylmaleimide. **** *p* < 0.0001; ** *p* < 0.01; * *p* < 0.05.

Bisindolylmaleimide (10 µM), a potent inhibitor of PKC, caused modest decreases in MCh-induced tension (2.7% ± 3.7%, *n* = 5) and F340/F380 (14.0% ± 6.6%, *n* = 5; Figure 7b, left panel), as did 3 nM glycopyrronium (7.2% ± 4.5% and 26.3% ± 4.0%, respectively; see Figure 3 and Figure 4). When glycopyrronium (3 nM) was applied with bisindolylmaleimide (10 µM), contraction was inhibited by 20.4% ± 10.7% and F340/F380 was reduced by 19.9% ± 9.7% (*n* = 5; Figure 7b, right panel). The relaxant effect of glycopyrronium was increased in the presence of bisindolylmaleimide, and the combination was more effective than the sum of the values for each agent (Figure 7b; *p* < 0.01). In contrast, the inhibitory effect of glycopyrronium on F340/F380 was not increased by bisindolylmaleimide, and was not greater in combination than the sum of the values for each agent (Figure 7b). In tension and F340/F380, the effects of adding bisindolylmaleimide to glycopyrronium were consistent with those of adding procaterol and salbutamol to glycopyrronium (see Figure 4 and Figure 5).

When procaterol (0.3 nM) and glycopyrronium (3 nM) were applied together in the presence of bisindolylmaleimide (10 µM), percent inhibition of tension was almost the same as in the absence of bisindolylmaleimide (Figure 7c). However, reduction of F340/F380 by procaterol with glycopyrronium in the presence of bisindolylmaleimide was markedly less than in its absence (*p* < 0.01; Figure 7c).

**Figure 8:**
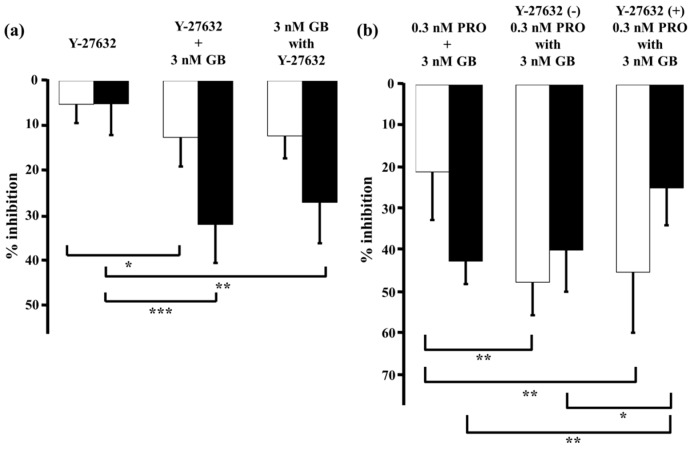
Y-27632-induced Ca^2+^ sensitization is not involved in synergy between β_2_-adrenoceptor agonists and muscarinic receptor antagonists. In each panel, percent inhibition of tension is represented by white columns, and F340/F380 is represented by black columns. (**a**) **Left**, percent inhibition of tension and F340/F380 with Y-27632 (1 µM); **Center**, sum of percent inhibition of tension and F340/F380 with Y-27632 (1 µM) and glycopyrronium (3 nM); **Right**, percent inhibition of tension and F340/F380 with combination of Y-27632 (1 µM) and glycopyrronium (3 nM); (**b**) **Left**, sum of percent inhibition of tension and F340/F380 with procaterol (0.3 nM) and glycopyrronium (3 nM); **Center**, percent inhibition of tension and F340/F380 with combination of procaterol (0.3 nM) and glycopyrronium (3 nM); **Right**, percent inhibition of tension and F340/F380 with combination of procaterol (0.3 nM) and glycopyrronium (3 nM) in the presence of Y-27632 (1 µM). GB, glycopyrronium bromide; PRO, procaterol; MCh, methacholine. *** *p* < 0.001; ** *p* < 0.01; * *p* < 0.05.

Y-27632 (1 µM), a selective inhibitor of Rho-kinase, caused small decreases in MCh-induced tension (5.1% ± 4.1%, *n* = 5) and F340/F380 (5.0% ± 6.8%, *n* = 5; Figure 8a; left panel). When glycopyrronium (3 nM) was applied with Y-27632 (1 µM), contraction was reduced by 12.0% ± 4.9% (*n* = 5) and F340/F380 by 26.5% ± 8.9% (*n* = 5; Figure 8a, right panel). The relaxant effect of glycopyrronium in the presence of Y-27632 was greater than without it, but the combined effect was not significantly greater than the sum of the effects of each agent (Figure 8a). Reduction in F340/F380 by glycopyrronium was also not significantly greater in the presence of Y-27632 than the sum of each agent’s effects (Figure 8a). Furthermore, the effect of adding Y-27632 to glycopyrronium on MCh-induced tension and F340/F380 was not consistent with that of adding procaterol and salbutamol to glycopyrronium (see Figure 4 and Figure 5). When procaterol (0.3 nM) was applied with glycopyrronium (3 nM) in the presence of Y-27632 (1 µM), inhibition of MCh-induced contraction was almost the same as in the absence of Y-27632 (Figure 8b). Percent inhibition of F340/F380 for procaterol with glycopyrronium in the presence of Y-27631 was markedly greater than in its absence (*p* < 0.05; Figure 8b).

### 2.5. Role of Large-Conductance Ca^2+^-Activated K^+^ Channels in Ca^2+^ Dynamics due to Procaterol and Salbutamol with Glycopyrronium

**Figure 9:**
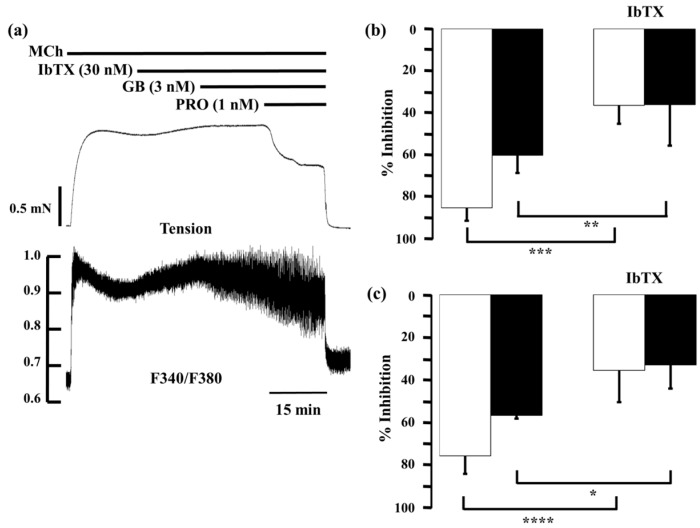
Involvement of large-conductance Ca^2+^-activated K^+^ channel in synergy between β_2_-adrenoceptor agonists and muscarinic receptor antagonists. (**a**) Typical record of tension (**upper side**) and F340/F380 (**lower side**) showing the inhibitory effect of a combination of procaterol (1 nM) and glycopyrronium (3 nM) in the presence of iberiotoxin (30 nM) on MCh (1 µM)-induced contraction; (**b**) Percent inhibition of tension (white column) and F340/F380 (black column) with combination of procaterol (1 nM) and glycopyrronium (3 nM) in the absence (**left**) or presence (**right**) of iberiotoxin (30 nM); (**c**) Percent inhibition of tension (whitecolumn) and F340/F380 (black column) with combination of salbutamol (10 nM) and glycopyrronium (3 nM) in the absence (**left**) or presence (**right**) of iberiotoxin (30 nM). IbTX, iberiotoxin; GB, glycopyrronium bromide; PRO, procaterol; SB, salbutamol; MCh, methacholine. **** *p* < 0.0001; *** *p* < 0.001; ** *p* < 0.01; * *p* < 0.05.

Described above, K_Ca_ channel activity plays an important role in the functional antagonism between β_2_-adrenoceptors and muscarinic receptors [22]. Therefore, we investigated whether K_Ca_ channel was involved in synergistic effect of LAMA and SABAs. In the presence of iberiotoxin, a selective antagonist of K_Ca_ channels, procaterol or salbutamol with glycopyrronium was applied to MCh-precontracted tissue (Figure 9a). Iberiotoxin (30 nM) caused modest increases in MCh-induced tension and F340/F380, to 111.5% ± 3.6% and 126.5% ± 13.4%, respectively. When procaterol (1 nM) was applied with glycopyrronium (3 nM) in the presence of iberiotoxin (30 nM), inhibition of contraction and F340/F380 were markedly decreased, to 34.7% ± 14.6% (*p* < 0.001) and 32.2% ± 10.8% (*p* < 0.01), respectively, compared to the values in the absence iberiotoxin (Figure 9b). Moreover, when salbutamol (10 nM) and glycopyrronium (3 nM) were applied in the same way, values of percent inhibition in contraction and F340/F380 by salbutamol (10 nM) with glycopyrronium (3 nM) in the presence of iberiotoxin (30 nM) were also markedly decreased to 36.5% ± 8.8% (*p* < 0.001) and 36.2% ± 19.6% (*p* < 0.05), respectively, compared to those values in the absence of iberiotoxin (Figure 9c).

## 3. Discussion

In the present study, GB (a LAMA) synergistically enhanced the inhibitory effect of procaterol and salbutamol (SABAs) on muscarinic contraction in airway smooth muscle via Ca^2+^ dynamics due to an inhibition in K_Ca_ channel activity, and Ca^2+^ sensitization due to an increase in PKC activity. These results demonstrate that this synergistic effect of combination between a SABA and a LAMA is mediated by the intracellular processes of β_2_-adrenoceptors and muscarinic receptors shown in Figure 1. We also observed similar synergistic effects between procaterol and tiotropium (another LAMA) in airway smooth muscle (Figure 6). These results demonstrate that pre-administration of a LAMA causes a marked augmentation in the relaxant action of SABAs, and that Ca^2+^ signaling due to both Ca^2+^ dynamics and Ca^2+^ sensitization contributes to this synergistic effect (cross talk between β_2_-adrenoceptors and muscarinic receptors). Recent reports have demonstrated that indacaterol, a LABA, synergistically augments the inhibitory effects of glycopyrronium against muscarinic contraction in airway smooth muscle [9,10]. Moreover, synergistic effects are similarly observed between olodaterol, another LABA, and tiotropium [38]. Therefore, synergistic effects against muscarinic contraction may be a ubiquitous phenomenon between β_2_-adrenoceptor agonists and muscarinic receptor antagonists.

Ca^2+^ signaling, mediated by Ca^2+^ dynamics via various channels and by Ca^2+^ sensitization due to the inactivation of MP via RhoA/Rho-kinase and PKC/CPI-17, contributes to airway smooth muscle tone induced by contractile agents via GPCRs, and other receptors [26,27,28,29,30,31,32,33,34,35,36,37,39,40,41,42,43]. The inhibitory effect of procaterol and salbutamol on MCh-induced contraction is mediated by reducing intracellular Ca^2+^ concentration; however, the reduction in contraction is greater than the reduction in intracellular Ca^2+^ concentration (Figure 2). These results are consistent with those shown in a previous report using isoprenaline, a SABA [43], suggesting that procaterol and salbutamol relax airway smooth muscle by reducing both Ca^2+^ dynamics and Ca^2+^ sensitization, like isoprenaline. Conversely, glycopyrronium inhibited MCh-induced contraction while also reducing intracellular Ca^2+^ concentration, although the contraction was reduced less than intracellular Ca^2+^ concentration, unlike the SABAs in this study (Figure 3). The relationship between tension and F340/F380 for glycopyrronium was consistent with that for SKF96365, a non-selective inhibitor of Ca^2+^ influx [43]. These results suggest that muscarinic receptor antagonists inhibit muscarinic contraction of airway smooth muscle by reducing Ca^2+^ dynamics. The role of Ca^2+^ signaling (dynamics and sensitization) in the inhibition of muscarinic contraction is not identical between β_2_-adrenoceptor activation and muscarinic receptor suppression (Figure 2 and Figure 3).

We also examined the effects of a combination of SABAs with glycopyrronium on MCh-induced tension and intracellular Ca^2+^ concentration. When the SABAs procaterol (0.1–1 nM) and salbutamol (1–10 nM) were applied in the presence of glycopyrronium, the relaxant effect was greater than the sum of the effects of the equivalent concentrations of each agent (Figure 4 and Figure 5), similar to the combined relaxant effect of indecaterol (a LABA) and glycopyrronium shown in previous reports [9,10]. These results demonstrate that pre-exposure to a lower concentration of glycopyrronium markedly enhances the effect of SABAs. To confirm these synergistic effects between SABAs and glycopyrronium, the inhibitory effect of these agents on tension and F340/F380 was evaluated using the BI theory, used previously to evaluate the synergistic effect of bronchodilators [44,45]. The main assumption of this model is that two or more agents act independently in terms of their sites of action [46,47]. The theory is represented by a very simple mathematical equation, and experimental data (from single points to entire dose–response curves) are applied to the equation to investigate synergy [12,46,47]. The relaxant effects of the SABAs procatrol (0.1–1 nM) and salbutamol (1–10 nM) in combination with glycopyrronium are greater than the expected values calculated by BI theory in equivalent concentrations of each agent (Figure 4 and Figure 5). These results demonstrate that the combination of SABAs with glycopyrronium synergistically inhibits muscarinic contraction. However, the reduction in F340/F380 ratio by procaterol (0.1–1 nM) and salbutamol (1–10 nM) in the presence of glycopyrronium is not greater than the sum of the effect of equivalent concentrations of each agent or the values calculated by BI theory in equivalent concentrations of each agent (Figure 4 and Figure 5). These results demonstrate that an inhibition in Ca^2+^ sensitization contributes to this synergistic effect of SABAs with LAMAs against muscarinic contraction in airway smooth muscle.

As described above, given that contractile agonists, which stimulate GPCRs, activate RhoA and PKC, this indicates that Ca^2+^ sensitization via RhoA/Rho-kinase or PKC/CPI-17 processes is involved in the mechanism underlying the synergistic effect between these receptors. The inhibitory effect of a combination of SABAs with glycopyrronium in tension and F340/F380 is mimicked by the addition of bisindolylmaleimide, a PKC antagonist, to glycopyrronium, but not by the addition of Y-27632, a Rho-kinase antagonist (Figure 5 and Figure 6). These results demonstrate that an inhibition in PKC-induced Ca^2+^ sensitization is involved in this synergistic effect, whereas Rho-kinase-induced Ca^2+^ sensitization is not. This phenomenon is similar to that observed in a previous report showing that the SABA isoprenaline acts by inhibiting Ca^2+^ sensitization independently of RhoA/Rho-kinase processes [43]. Because muscarinic receptor agonists act on PKC, sensitivity to intracellular Ca^2+^ is augmented via activation of CPI-17, independent of RhoA/Rho-kinase processes. Activation of β_2_-adrenoceptors by binding of agonists such as SABA causes an increase of PKA activity. CPI-17 may be inhibited by PKA-induced phosphorylation. As a result, PKC-induced Ca^2+^ sensitization is attenuated by PKA [48]. Although it is still unclear about involvement of β_2_-adrenergic action in PKC-induced Ca^2+^ sensitization mediated by contractile agonists in airway smooth muscle, a recent report has demonstrated that PKA suppresses (phosphorylates) CPI-17 in endothelial cells [49]. This finding may support our data that β_2_-adrenergic relaxation is mediated by an inhibition of PKC-induced Ca^2+^ sensitization. Hence, PKC could be a target protein for the cross talk based on Ca^2+^ sensitization between β_2_-adrenoceptors and muscarinic receptors. From results of our study, SABA may attenuate PKC-induced Ca^2+^-sensitization via activation of PKA. To clarify this phenomenon, further investigation will be needed.

Although muscarinic receptor activation causes contraction by increasing both Ca^2+^ dynamics and Ca^2+^ sensitization [26,43], muscarinic blockade is mediated by affecting Ca^2+^ dynamics (reducing intracellular Ca^2+^ concentration), not by affecting Ca^2+^ sensitization (Figure 3). Hence, muscarinic receptor antagonists might enhance the relaxant effects of SABAs via mechanisms other than muscarinic blockade-induced Ca^2+^ dynamics. LAMAs may act on β_2_-adrenoceptors via PKC-induced Ca^2+^ sensitization, leading to this synergistic effect between these two receptors [38].

Given that K_Ca_ channel activity plays an important role in the functional antagonism between β_2_-adrenoceptors and muscarinic receptors [22], intracellular processes concerning these channels may be involved in this synergistic effect [17,18,20,22,50]. The G protein/K_Ca_ channel processes may contribute to an increase in response to muscarinic receptor agonists (airway hyperresponsiveness) [22,23] and a decrease in response to β_2_-adrenoceptor agonists (β_2_-adrenoceptor desensitization) [51,52]. We examined involvement of these processes in this synergistic effect. In the presence of iberiotoxin, a selective K_Ca_ channel inhibitor, the relaxant effects of the SABA/glycopyrronium combination were markedly attenuated with an increase in F340/F380 (Figure 7). As shown in Figure 4, Figure 5 and Figure 6, Ca^2+^ dynamics might not be involved in the synergistic effect between SABAs and glycopyrronium. However, these results indicate that a reduction in intracellular Ca^2+^ concentration by K_Ca_ channel activation also causes this synergistic effect between these two receptors (Figure 1), consistent with the combination of indacaterol with glycopyrronium [9,10]. Because the outward currents are markedly increased by K_Ca_ channel opening, the membrane potential is hyperpolarized, leading to decreasing voltage-dependent Ca^2+^ entry (VDCE), such as L-type voltage-dependent Ca^2+^ channels [9,10,23] (Figure 1). Therefore, an inhibition in the G_i_/K_Ca_ channel inhibitory linkage and an augmentation in the G_s_/K_Ca_ channel stimulatory linkage are thought to be another functional intracellular mechanism of this synergistically relaxant effect of combination of β_2_-adrenoceptor agonists with muscarinic receptor antagonists. K_Ca_ channels also could be a target protein for the cross talk based on Ca^2+^ dynamics between these two GPCRs.

## 4. Experimental Section

### 4.1. Tissue Preparation and Bathing Solution

Tracheae were excised from male Hartley guinea pigs (300–350 g) after intraperitoneal injection of pentobarbital (150 mg/kg). The tracheal rings were opened by cutting longitudinally at the cartilaginous region, and the epithelium was peeled off carefully. Normal bathing solution (in mM: NaCl 137, KHCO_3_ 5.9, CaCl_2_ 2.4, MgCl_2_ 1.2, and glucose 11.8) bubbled with a mixture of 99% O_2_ and 1% CO_2_ (pH 7.4) was perfused at a constant flow of 4 mL/min. Organ bath temperature was maintained at 37 °C. The animal protocols used in this work were evaluated and approved by the Research Center for Animal Life Science Committee, Shiga University of Medical Science (identification code: 2013-4-1, 15 April 2013).

### 4.2. Isometric Tension Recording and Measurement of Fura-2 Fluorescence

This was conducted as described previously [26,43,53]. In brief, muscle strips were incubated with 15 µM fura-2/AM for about 2 h at room temperature (22–24 °C). The non-cytotoxic detergent Pluronic F-127 (0.01% *w*/*v*) was added to increase the solubility of fura-2/AM. After the fura-2/AM had penetrated the tissue, the experimental chamber was filled with normal bathing solution. Isometric tension and fura-2 fluorescence of muscle strips were measured simultaneously, using a displacement transducer and a spectrofluorometer (CAF-110, Japan Spectroscopic, Tokyo, Japan). The mucosal side of the smooth muscle was exposed to excitation light, and the intensities of excitation fluorescence at 340 nm and 380 nm were measured after background subtraction. Because the dissociation constant of fura-2 for Ca^2+^ in smooth muscle cytoplasm was shown to be different from that measured in vitro, the absolute amount of intracellular Ca^2+^ concentration was not calculated [54]. Therefore, the ratio of F340 to F380 (F340/F380) was used as a relative indicator of intracellular Ca^2+^ concentration. Methacholine (MCh, 1 µM) was administered for 5 min at 10 min intervals until the control response to 1 µM MCh was established, and then the experiment was started. Muscle tension and F340/F380 in the resting state were taken as 0%, and the value of percent inhibition of tension and F340/F380 were measured taking the control response to MCh (1 µM) as 100%. Indomethacin (2 µM) was administered throughout the experiments to abolish resting tone.

### 4.3. Experimental Protocol

To examine the relationship between muscle tone and intracellular Ca^2+^ concentration in the presence of β_2_-adrenoceptor agonists or muscarinic receptor antagonists, procaterol (0.1–10 nM), salbutamol (1–100 nM) or glycopyrronium (0.3–10 nM) were each administered cumulatively to tissue precontracted with MCh (1 µM throughout the study). To examine this relationship in the presence of a combination of β_2_-adrenoceptor agonists and muscarinic receptor antagonists, procaterol (0.1–1 nM) and salbutamol (1–10 nM) were added to MCh-precontracted tissue in the presence of glycopyrronium (3 nM), and procaterol (0.3–1 nM) was added to MCh-precontracted tissue in the presense of tiotropium (1 nM). To investigate the role of Ca^2+^ dynamics due to K_Ca_ channel activity in the synergistic effects of β_2_-adrenoceptor agonists and muscarinic receptor antagonists, procaterol and glycopyrronium were added together to MCh-precontracted tissue, in the presence of iberiotoxin (30 nM), a selective inhibitor of K_Ca_ channels, which are densely distributed on airway smooth muscle cell membrane [55]. Moreover, to investigate the role of Ca^2+^ sensitization due to Rho-kinase and PKC in the synergistic effects of β_2_-adrenoceptor agonists and muscarinic receptor antagonists, procaterol and glycopyrronium were both added to MCh-precontracted tissue in the presence of Y-27632 (1 µM), a selective inhibitor of Rho-kinase, and bisindolylmaleimide (10 µM), a potent inhibitor of PKC. Each reagent at each concentration was administered for 15 min (except MCh). Time-matched control tissues were treated similarly to the test tissues, but exposed continuously to the normal bathing solution (sham incubation) instead of procaterol, salbutamol, iberiotoxin, Y-27632, or bisindolylmaleimide.

### 4.4. Analysis of Synergistic Effect

The synergistic effect of glycopyrronium and procaterol or salbutamol and that of tiotropium and procaterol on contracted airway smooth muscle and change in intracellular Ca^2+^ concentration was evaluated using the BI theory. This model assumes that two or more agents act independently, with different modes and sites of action [46], and is expressed by the following equation:

E(*x, y*) = E(*x*) + E(*y*) − E(*x*) × E(*y*) (1)

where E is the fractional effect, and *x* and *y* are the doses of two compounds in a combination experiment. If the combined experimental value is higher than the expected value, the interaction is synergistic. If it is lower, the interaction is antagonistic [46,47].

### 4.5. Materials

MCh, indomethacin, procaterol, salbutamol, and glycopyrronium were obtained from Wako pure Chemical Industries (Osaka, Japan). iberiotoxin was obtained from Peptide Institute (Osaka, Japan). Tiotropium, bisindolylmaleimide and Y-27632 were obtained from Sigma (St. Louis, MO, USA). Fura 2-AM was obtained from Dojin Laboratories (Kumamoto, Japan).

### 4.6. Statistical Analysis

All data are expressed as the mean ± standard deviation (SD). Statistical significance was assessed by *t*-test or Student’s unpaired *t*-test and one-way analysis of variance. *p* < 0.05 was considered significant. Statistical analyses were performed using JMP version 11 (SAS Institute Inc., Cary, NC, USA).

## 5. Conclusions

**Figure 10:**
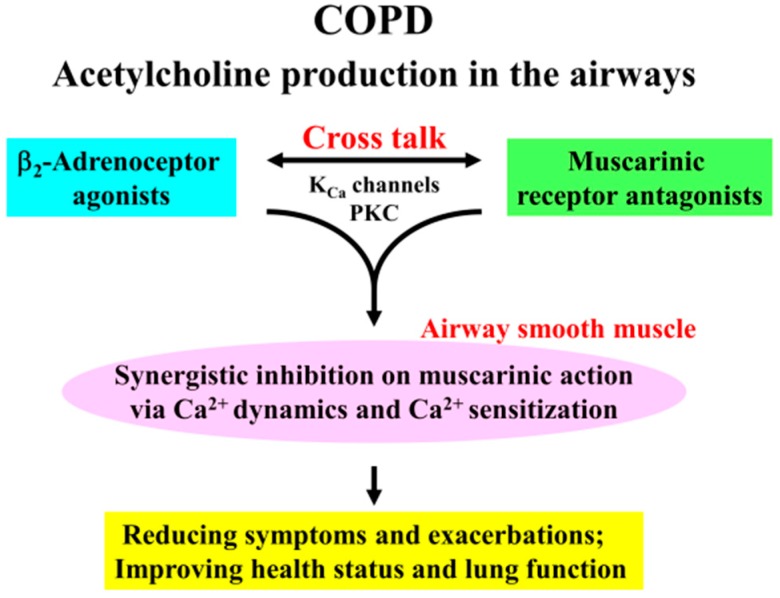
Clinical effectiveness of Combination of β_2_-adrenoceptor agonists and muscarinic receptor antagonists is in the treatment for COPD via Ca^2+^ signaling (Ca^2+^ dynamics and Ca^2+^ sensitization). Since addition of β_2_-adrenoceptor agonists to muscarinic receptor antagonists markedly enhance an inhibition of airway smooth muscle contraction, combination of these two agents are useful to suppression of excessive stimulation to muscarinic receptors induced by acetylcholine production in the airways, which is an fundamental characteristic for COPD. This phenomenon is due to cross talk between these two receptors via K_Ca_ channel-induced Ca^2+^ dynamics and PKC-induced Ca^2+^ sensitization in airway smooth muscle. Therefore, this combination therapy leads to reducing symptoms such as dyspnea on exertion and frequency of exacerbations, and to improving health status and lung function in patients with COPD.

The combination of β_2_-adrenoceptor agonists with muscarinic receptor antagonists causes a synergistic inhibition of muscarinic contraction in airway smooth muscle. Ca^2+^ signaling via PKC-induced Ca^2+^ sensitization and K_Ca_ channel-induced Ca^2+^ dynamics play a functional role in this synergistic effect (Figure 1). K_Ca_ channels and PKC are key protein for cross talk between β_2_-adrenoceptors and muscarinic receptors (Figure 10). Since it is generally considered that acetylcholine production (both neural and non-neural) in the airways is an essential pathophysiology for COPD, the effectiveness of therapy for this disease is depend on inhibitory action against excessive muscarinic stimulation. Hence, combination between these two agents is beneficial to reducing symptoms and exacerbations, and to improving health status and lung function (Figure 10). Our results provide further evidence that the combination of β_2_-adrenoceptor agents and anti-cholinergic agents are a promising advancement in bronchodilator therapy for COPD and asthma, and that PKC and K_Ca_ channels may be novel targets for bronchodilator research and development.
